# When do we need to take action? Cut-off values for the hamburg inventory for measuring quality of life in oncological patients (HELP-6)

**DOI:** 10.1186/s41687-026-01198-8

**Published:** 2026-09-19

**Authors:** Theresa Schrage, Mirja Görlach, Christian Stephan Betz, Carsten Bokemeyer, Nicolaus Kroeger, Volkmar Mueller, Andreas Krüll, Holger Schulz, Christiane Bleich

**Affiliations:** 1 Department of Medical Psychology, University Medical Center Hamburg-Eppendorf, Hamburg, Germany; 2 Department of Otorhinolaryngology, University Medical Center Hamburg-Eppendorf, Hamburg, Germany; 3 Department of Internal Medicine II, University Medical Center Hamburg-Eppendorf, Hamburg, Germany; 4 Department of Stem Cell Transplantation, University Medical Center Hamburg-Eppendorf, Hamburg, Germany; 5 Department of Gynecology, University Medical Center Hamburg-Eppendorf, Hamburg, Germany; 6 Department of Radiotherapy and Radiation Oncology, University Medical Center Hamburg-Eppendorf, Hamburg, Germany

**Keywords:** Cut-off values, Health-related quality of life, Psychooncology, Receiver operating characteristics analysis, Patient-reported outcome measurement, Routine care

## Abstract

**Purpose:**

Results of patient-reported outcome measurements need to be comprehensible and conclusive for a successful integration into treatment in cancer care. The aim of this study was to determine cut-off values for the recently developed “Hamburg Inventory for Measuring Quality of Life in Oncological Patients-6” (HELP-6), a short measurement for Health-related Quality of Life (HrQoL) in routine care in oncological patients.

**Methods:**

Oncological in- and out-patients with differing cancer entities participated in this study. The survey included the HELP-6 and standardized questionnaires (Distress-Thermometer, PHQ-4, Fact-G, PDI-G). Six receiver operating characteristics analyses were performed, one for each scale of the instrument (Emotional Health, Physical Ailment, Social Functionality, Autonomy, Dignity, Resources).

**Results:**

For five of the six scales cut-offs could be determined. Areas under the Curve were between 0.7 and 0.8. However, the Dignity scale demonstrated poor discriminative validity, failing to distinguish adequately between severity levels of patient groups.

**Conclusion:**

By applying the HELP-6 with the cut-off values established in this study, clinicians can more readily interpret patients’ HrQoL, thereby supporting effective clinical decision-making. This feature is especially valuable in routine care, where streamlined monitoring of patient quality of life is paramount. Further conceptualization and validation of the Dignity scale, as well as its integration into patient-reported outcome measures, are warranted.

**Trial registration:**

The complete study including the development of the instrument was registered at Open Science Framework ***redacted for peer-review***. Clinical trial number: not applicable.

**Supplementary Information:**

The online version contains supplementary material available at https://doi.org/10.1186/s41687-026-01198-8.

## Abstract (plain language summary)

Recently, a new questionnaire was developed to measure quality of life in patients with cancer. This questionnaire is intended for the use in clinical practice, where it needs to be easy to interpret. To achieve this, clinicians must be able to distinguish between patients with impaired quality of life and those without.

For this reason, we aimed to establish cut-off values that clearly separate these two groups – patients with affected quality of life and those without. Using these cut-off values, clinicians can quickly identify which patients may benefit from additional support or a referral to a psychooncologist.

We performed statistical analyses to calculate these cut-off values. The results show, that for five of the original six questions of the questionnaire, the cut-off values successfully differentiate between these two patient groups. However, for one question, the results indicate that no useful cut-off value could be found.

**Supplementary Information:**

The online version contains supplementary material available at https://doi.org/10.1186/s41687-026-01198-8.

## Objectives

Patient-reported outcomes (PROs) integrate patients’ perspectives on their mental and physical health into their treatment. An effective way of using and implementing PROs is through patient-reported outcome measurements (PROMs). These measuring tools are utilized with greater frequency, particularly in the field of oncology [1–4]. Practical implications of elevated PROM results are essential for clinicians to benefit from and effectively integrate these results in their work [5].

Implementing PROMs in routine (cancer) care has shown to have positive effects on survival, symptoms such as pain, anxiety and depressive symptoms, psychosocial distress, health-related quality of life (HrQoL), shared-decision making, and in general, patient satisfaction [1, 6, 7]. The application of PROMs as electronic health technologies also is employed more frequently and has the advantage of improving data quality, leads to similar or faster completion time, and facilitates symptom management [3].

When dealing with PROMs in routine care certain conditions must be taken into consideration. PROMs should take up little completion time, should be easily comprehensible, evaluation should be simple and results interpreted easily, and additionally, feedback should be given to patients [1, 8–11]. A key issue is the usability of the PROs for clinicians. Without this they will not be able to use the information during treatment and thereby provide feedback to the patient or adjust therapy. Among others, a clear reference point, a cut-off, of a PROM-scale helps clinicians discriminate between critical cases with a need for additional support and non-critical cases [12]. Also, national guidelines for psycho-oncological diagnostics, counseling, and treatment of adult cancer patients recommend that cut-off values should be available for the PROMs used [13, 14].

Within the project PRO-Onko-Routine, the questionnaire “Hamburg Inventory for Measuring Quality of Life in Oncological Patients-6” (HELP-6) was developed [15]. In a first step, a qualitative study was performed, conducting qualitative interviews with oncological patients, focus groups with oncological clinicians, and an expert discussion to identify and determine important dimensions of HrQoL in oncological routine care [16]. Second, the resulting questionnaire was psychometrically evaluated in an observational study. One result is, that the number of the questionnaires items was reduced from ten to six items, resulting in the final version: the HELP-6 [17]. The questionnaire consists of 6 continuous one-item scales ranging from zero to ten. The final six scales are: Emotional Health, Physical Ailment, Social Functionality, Autonomy, Dignity, and Resources. The design of the scales is similar to that of the NCCN Distress Thermometer (DT) which has a cut-off of ≥ 5 for the German population [18]. The HELP-6 is intended for continuous monitoring in the routine care of oncological patients. With six separate interpretable scales, the HELP-6 is shorter than other instruments developed for research purposes, while also providing more information about patients with cancer than generic instruments. In clinical practice it is essential to balance the need for information with the shortage of time. There was a gap of HrQoL instruments for this exact use case which the HELP-6 was developed to fill. However, to be more accessible, the information from the questionnaire needs to be easier to interpret.

As the clinical implications are important for the use of a PROM in routine care, it is necessary to define cut-offs for the HELP-6 questionnaire. The aim of this analysis was to determine cut-off values for the recently developed HELP-6, a short measurement for HrQoL in routine care in oncological patients.

## Research approach

The study is part of the project “PRO-ONKO-Routine” [15], succeeding the studies of the instrument development and the determination of the psychometric properties. The study protocol has been published elsewhere [15], as has the study protocol for implementation analysis [19]. Funding for this project was granted by ***redacted for peer-review*** and pre-registered at OSF – Open Science Framework [20] ***redacted for peer-review***. This study was reported in accordance with the STROBE statement [21].

### Study design

This observational study enrolled oncological in- and outpatients at ***redacted for peer-review***. Data were collected at two points of measurement in five departments of the ***redacted for peer-review***: the Department of Internal Medicine II, the Department of Stem Cell Transplantation, the Department of Gynecology, the Department of Radiotherapy and Radiation Oncology and the Department of Otorhinolaryngology. The five departments are members of the University Cancer Center ***redacted for peer-review***. The local research ethics committee of the ***redacted for peer-review*** approved the study design (no. ***redacted for peer-review***). All participants provided written informed consent. The questionnaire was presented to the participants in paper-pencil format. Data collection started on June 1, 2018, and ended on February 28, 2019.

### Measurements

Additional to the HELP-6 [17] a set of established standardized measures was included in the quantitative survey at both points of measurement. The socio-demographic questionnaire includes questions on age, gender, education, employment status, marital status, children, and living situation. The survey was presented to the patients in paper-pencil format. In the long term, however, the questionnaire was and will be used digitally.

The following standardized questionnaires were included in the survey:

#### Fact-G

In order to assess HrQoL we included the Functional Assessment of Cancer Therapy – General (Fact-G) [22]. It measures four dimensions of well-being (physical, social, emotional, and functional) with a total of 27 items. Subscales range from 0 to 24/28, with a higher score indicating better quality of life [22]. In this study, the cut-off values of the subscales physical, social, and functional well-being will be used. Physical well-being has a cut-off of ≤ 15, the subscale social well-being has a cut-off of ≤ 16, and functional well-being has a cut-off of ≤ 11 [23].

#### Distress-Thermometer

Additionally, the distress scale of the Distress-Thermometer (DT) [18] often used in oncological practice was included. The distress scale measures distress from 0 (“no distress”) to 10 (“extreme distress”). The cut-off for the German population is ≥5 [18].

#### PDI-G

In our survey, dignity was assessed by using the Assessment of patients’ dignity in cancer care (PDI-G) [24]. With 25 items the PDI-G measures the most common factors related to a persons’ sense of dignity. A high score represents a greater problem regarding factors of dignity [24]. So far, no cut-off values have been determined for the PDI-G.

Moreover, medical data (cancer entity, treatment, and treatment as in- or outpatient) were retrieved from the electronic patient record.

### Recruitment of participants

Inclusion criteria for patient recruitment were: age of 18 years or more, cancer diagnosis, sufficient German language skills, and no severe cognitive or verbal impairments that would affect their ability to give informed consent. Potential inpatients were identified by medical staff and addressed by research staff. In the outpatient departments, the oncological patients were approached directly by research staff. Outpatients were interviewed once during their treatment and again one week later. Inpatients were interviewed at the beginning of their treatment and three to seven days later.

### Methodological approach

**Fig. 1:**
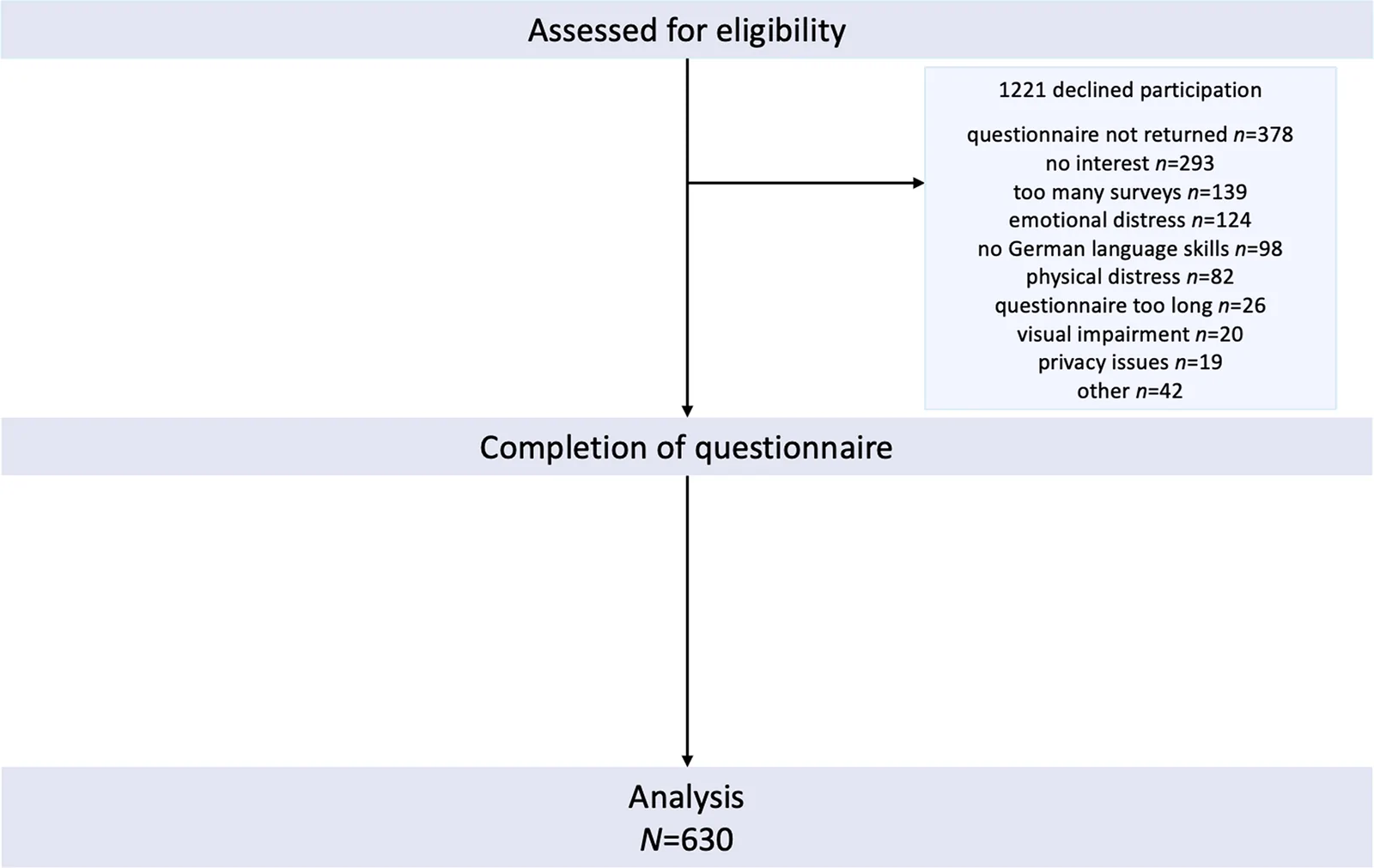
Flow chart

Descriptive statistics using frequencies and mean values with standard deviations were computed to describe the sample. Because of missing values ranging from 30% and more, *n* = 9 cases were excluded from the sample description (Fig. 1). All analyses were performed using complete cases only. Missingness by variables used for the analysis is depicted in the supplements (Table S1). To test the robustness of the handling of missing data, a sensitivity analysis was conducted. Instead of using exclusively complete cases, the missing data were estimated using the expected-maximization algorithm.

To enhance the comprehensibility of the results, all invers HELP-6 scales (Social Functionality, Dignity, Resources) were inverted. Consequently, an elevated value on each scale can be associated with an impaired HrQoL.

In preparation of the Receiver Operating Characteristics (ROC) analysis, the internal grouping of our data was generated according to external criteria. These criteria were derived from the standardized questionnaires DT, FACT-G, and PDI-G [18, 22, 24, 25]. In case of the DT and FACT-G, published cut-offs were used to perform the internal formation of four of the six HELP-6 scales (see Table 3). Since no cut-offs are known for the PDI-G, we applied a content criterion to discriminate between the groups. The items of the PDI-G are assigned values ranging from one to five. They are defined as: no problem, a small problem, a problem, a great problem, an overwhelming problem. We grouped participants rating “a problem” and higher in one group and participants rating “a small problem” and lower in a second group. Prior to the analyses the three scales of the HELP-6 social functionality, dignity, and resources were recoded so that for every scale a higher value is associated with a lower HrQoL.

To generate cut-offs for the HELP-6 scales six ROC analyses were performed. Criteria to determine the cut-offs were Area Under the Curve (AUC) and Youden’s index [26]. The AUC indicates how well a questionnaire can discriminate between groups. The values range from 0.5 to 1.0, where 0.5 refers to a discrimination ability of a 50% chance and 1.0 refers to a test with perfect discrimination [27]. The second criterion is Youden’s index which defines as follows: sensitivity + specificity – 1 [26]. The index has values that range from 0 (no diagnostic value) to 1 (perfect test). In a ROC curve, 1 is depicted in the upper left corner. In case of an index of 1, the sensitivity and the specificity both equal 1, so there are no false positives or false negatives.

The interpretation of both the AUC and the Youden-index is not entirely conclusive, as these values have to be interpreted regarding the context. An orientation is given for the AUC as that values of 0.7 to 0.8 are acceptable, 0.8 to 0.9 and above are excellent to outstanding [27, 28]. Where values are below 0.7 the scales’ ability to discriminate between the correctly tested cases should be viewed critically. In this study, no cut-offs will ultimately be determined if the AUC is under 0.7. To determine a cut-off for the scales, the Youden-index was maximized. When the Youden’s index indicated a cut-off with a sensitivity below 0.70, the cut-off with the closest threshold and a sensitivity above 70% was selected [29].

## Findings

### Sample characteristics

An extensive sample description can be found in ***redacted for peer-review***. *N* = 630 participants were included in the analyses (Table 1). The sample had a mean age of 59.2 years (*standard deviation (SD)* = 13.63), with 56% female participants. Nearly half of the participants had a low education (45.1%) and most of them were employed (43%) or retired (34%). About two thirds lived in a permanent relationship (71%), lived with a partner, parents, and or children (75%). Cancer-related patient characteristics could only be retrieved form medical records for part of the participants (*n* = 479 (75.01%)). Of these, most patients were diagnosed with breast cancer (*n* = 125, 27.8%), prostate cancer (*n* = 47, 10.5%), or larynx cancer (*n* = 32, 7.1%). Most of the participants were outpatients (*n* = 339, 72.4%). Most often, they received chemotherapy (*n* = 302, 63.0%) and/or surgery (*n* = 300, 62%).

**Table 1:** Sample characteristics^a^

	Total sample *N* = 630
**Age **^**b**^, mean (SD)	59.21 (13.63)
**Gender**	
Female	355 (56.3%)
Male	275 (43.7%)
**Education** ^**c**^	
Low	284 (45.1%)
Medium	131 (20.8%)
High	207 (32.9%)
**Employment relationship**	
Employed	271 (43.1%)
Self-Employed	49 (7.8%)
Apprentice/Student	10 (1.6%)
Homemaker	24 (3.8%)
Unemployed or incapacitated for work	61 (9.7%)
Retired	215 (34.1%)
**Relationship status**	
Without permanent relationship	177 (28.1%)
Married or in a permanent relationship	453 (71.9%)
Number of **children**, mean (*SD*)	1.27 (1.05)
**Living situation**	
Alone	157 (24.9%)
Living with partner/children/parents	473 (75.1%)
**Residence**	
Village	90 (14.3%)
Small city (< 25,000 residents)	87 (13.8%)
Medium city (25,000 to 100,000 residents)	76 (12.1%)
Large city (> 100,000 residents)	377 (59.8%)

^a^ The table “Sample characteristics” was first published in Schrage et al. [17]

^b^ age in years

^c^ low = graduation after 9 or 10 years of school, medium = graduation after 12 or 13 years of school, high = graduation after more than 13 years of school (e.g. colleague or university)

Descriptive statistics for the HELP-6 scales of this sample are displayed in Table 2. The means for the three scales Emotional Health, Physical Ailment, Autonomy ranged from 4.1 to 5.5, the means for the other three scales (Social Functionality, Dignity, and Resources) were lower with 1.2 to 2.5. In summary, a high value is associated with higher levels of impairment in HrQoL patients showed higher impairment in Emotional Health, Physical Ailment, and Autonomy. Two scales had deviating values of either skewness (Social Functionality) or kurtosis (Dignity).

**Table 2:** Descriptive statistics for the HELP-6 scales

HELP-6 scales	*N*	mean	SD	skewness	kurtosis
Emotional health	626	5.5	2.9	-0.1	-1.1
Physical ailment	625	4.1	2.7	0.3	-0.8
Social functionality	627	1.5	2.1	1.8	2.8
Autonomy	623	4.4	2.9	0.3	-0.9
Dignity	621	1.2	1.6	2.1	6.0
Resources	624	2.5	2.1	0.9	0.7

### Receiver operating characteristics curves of the HELP-6 scales

**Fig. 2:**
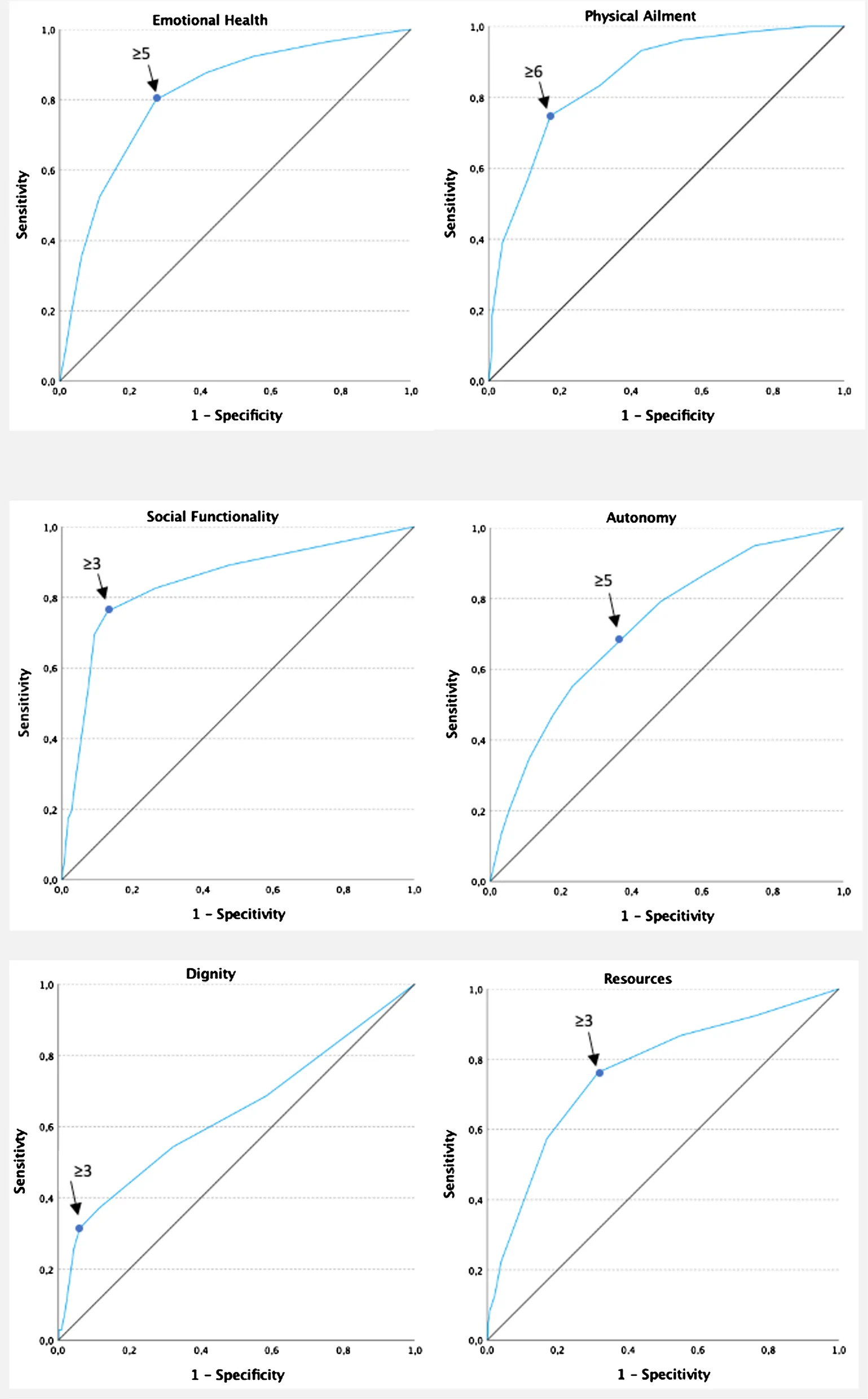
Receiver operation characteristic curves for the HELP-6 scales. The blue line displays the ROC curve and plots all potential cut-offs of a test or questionnaire (sensitivity against 1-specificity), whereas the diagonal line displays chance-level discrimination (AUC = 0.50). The point highlighted on the blue line highlights the optimal cut-off for that test

The Area under the Curve across all scales ranged from 0.64 to 0.86, with a Youden statistics of 0.31 to 0.64. In all cases except for one, sensitivity was above 70% and specificity above 61%. The optimal cut-offs determined for each scale ranged from ≥ 3 to ≥ 6. More detailed information is displayed by the summary of results in Table 3 and the Receiver Operating Characteristics Curves in Fig. 2.

**Table 3:** Summary of results from receiver operating characteristics analyses

HELP-6 scale	Comparative scale	Sample size	Area under the Curve (95% CI)	Youden-Index	Optimal cutoff	Sensitivity (%)	Specificity (%)	Positive likelihood ratio	Negative likelihood ratio	Positive predictive value (%)	Negative predictive value (%)
Emotional Health	Distress scale, *DT*	617	0.81 (0.78–0.85)	0.53	≥ 5	80%	73%	2.96	0.27	76	77
Physical Ailment	Physical well-being, *Fact-G*	574	0.86 (0.78–0.91)	0.57	≥ 6	75%	83%	4.41	0.30	56	91
Social Functionality	Social well-being, *Fact-G*	545	0.84 (0.82–0.89)	0.63	≥ 3	76%	87%	5.85	0.28	32	77
Autonomy	Loss of autonomy, *PDI-G*	613	0.72 (0.68–0.76)	0.31	≥ 5	70%	61%	1.79	0.49	46	81
Dignity	Loss of sense of worth and meaning, *PDI-G*	582	0.64 (0.52–0.75)	0.64	≥ 3	37%	89%	3.36	0.71	17	96
Resources	Functional well-being, *Fact-G*	574	0.76 (0.72–0.81)	0.45	≥ 3	76%	68%	2.38	0.35	44	90

### Sensitivity analysis

The ROC analyses using the data set with all missing values imputed confirmed the results of the complete-case analyes. The choice of optimal cut-offs remained the same as in the main analyes and the AUC and confidence intervals achieved nearly identical results. Results of the sensitivity analysis are displayed in the supplementary material (Table S2).

### Interpretation

### Performance of HELP-6 scales

The determination of cut-offs within the health-related quality of life measurement HELP-6 was designed to enable straightforward and rapid interpretation of results, thereby facilitating support in the decision-making process for PROM evaluation. We were able to select cut-offs for five measurement scales by conducting ROC analyses: Emotional Health, Physical Ailment, Social Functionality, Autonomy, and Resources. The criteria AUC, Youden’s statistic, and sensitivity and specificity indicate a good ability to discriminate between the positive and negative respondents. Consequently, the HELP-6 demonstrates adequate discriminatory capacity in differentiating among between patients who are more or less affected in these aspects of their HrQoL. This is of relevance insofar as it mirrors the outcomes of widely used domains of standardized HrQoL measurements. Further, especially the determination of a cut-off for the domain Resources is essential for the application in routine care. After patients and clinicians alike emphasized the importance of a mechanism to identify patients’ need for support, this specific domain was added to the questionnaire [16]. With an included cut-off, the domain’s capacity to assist clinicians in their decision-making is facilitated.

### Challenges with autonomy and dignity

However, the ROC analysis of the scale Autonomy resulted in a comparatively low Youden-index and an AUC of just above 0.7. As well, the sensitivity was just about 70%. A cut-off of ≥ 5 could be determined but the discriminative ability is not definite and should be investigated in further studies. In summary, this indicates that the scale does not adequately distinguish between patients with impaired autonomy and those without. The concept of autonomy is not universally defined, and chronic illness further complicates the definition of this construct. This is because patients are people, people with worldviews and perceptions of autonomy that they developed before becoming chronically ill. When faced with a chronic illness, these state-like perceptions of autonomy can be complemented or reshaped by trait-like changes. These changes may be influenced by restrictions in bodily functions or decision-making experiences during treatment. Chronic illness does not necessarily equate to impaired autonomy, but it can render autonomy more volatile. Consequently, it can be more difficult to capture and categorize using simple binary classifications.

Furthermore, no cut-off could be selected for the scale Dignity. Several criteria indicate that based on the ROC analysis of the Dignity scaleno sufficient discrimination between the two groups of correctly identified positive and negative cases could be achieved. The first indicator of this is a low AUC of < 0.7. Additionally, the sensitivity associated with the highest Youden’s statistics was 37%. Choosing another threshold would not have elevated the sensitivity above 70%. The sensitivity indicates the percentage of correctly identified positive cases. In case of this study, these are correctly identified cases with compromised HrQoL. The intention of the HELP-6 is that Health Care Professionals should be supported in recognizing patients’ low or impaired HrQoL (identifying true positives). At the same time, the costs of addressing patients regarding their HrQoL even though they are not distressed or do not need support (identifying false positives) are low and probably have no negative effect. Therefore, sensitivity of the HELP-6 scales is more important and should have a higher percentage than specificity. In addition, the positive predictive value was 17% meaning that in case of a positive test result only 17% of the participants will truly show elevated impairment in their feelings of dignity. In this study, the Dignity scale was found to have no discriminatory ability that would be useful for clinical practice regarding these criteria.

The results for the Dignity scale are not entirely unexpected and are in line with the results of the previous validation analysis for this scale [17]. The distribution of the scale Dignity had high kurtosis and skewness because on average patients rated their perceived dignity to be very high. We can only speculate whether this is a phenomenon related to an overall high quality of care at the university hospital and certified cancer center. Another reason for this result could be the conceptualization of dignity. Based on a previously conducted qualitive study with patients, Health Care Professionals, and other experts, dignity was understood as individualism and as respect for patients’ boundaries and privacy [16]. This differs from the model of perceived dignity in terminally ill patients developed by Chochinov and colleagues [30]. The authors conclude three categories of which one includes associations with the existing physical illness, a second category includes associations to socially mediated factors, and a third associates to psychological concerns like autonomy and continuity of self [30, 31].

Furthermore, even though autonomy and dignity are not new concepts in cancer research, they have rarely been included in HrQoL measurements. More research is needed on how to incorporate these concepts into patient-reported measurements in routine clinical oncology settings. In this study, the discrimination of groups with higher and lower perceived autonomy and dignity have been based on the external criterion of the dignity questionnaire PDI-G [24]. So far, no cut-off values have been determined for the PDI-G and to the best of our knowledge, no other dignity questionnaire for this population of cancer patients is available. The content criterion we applied is not empirically tested and therefore is not completely reliable. This study can be viewed as a starting point for further testing on dignity and autonomy in general and regarding cut-offs.

### Clinical implications

The clinical advantage of established cut-offs is mainly the ease and speed of interpretation and support in decision-making. In addition to cut-off’s, predefined and documented recommendations for action are needed to complement the practical implications of a PROM for continuous assessment in routine care. Furthermore, when used to monitor HrQoL, it can be argued that repeated measurement within patients allows a trend in the data to be identified. This trend could also support interpretation of the patients HrQoL and guide treatment decisions. However, the length of in- and outpatient stays, and therefore the possible points of measurement, vary. While some patients may have long stays or many appointments, others may have short stays or few contacts with a health care institution. Therefore, providing cut-offs in addition to visualization of HrQoL trajectories is beneficial.

In case of the HELP-6, this means that clinicians could initiate an intervention if a patient has an elevated result. Regarding the specific implications for the HELP-6 scales, the Resources scale should be the gatekeeper in determining whether action by Health Care Professionals is warranted. For instance, a patient may score highly on the Physical Ailment scale due to pain, but if this symptom is already being treated and the patient’s Resources score is not elevated — indicating adequate coping resources — no further action may be warranted. Conversely, if Resources scores are elevated, results on the other four scales of Emotional Health, Physical Ailment, Social Functionality, and Autonomy can guide the direction of necessary interventions. In general, this may prompt further clarification of the specific issue with the patient. More specifically, they could lead to a consultation with a psychooncologist (Emotional Health scale), referral to paint management or physiotherapy (Physical Ailment scale), a consultation with a social worker (Social Functionality Scale), or targeted adjustments to improve a patient’s perceived autonomy, such as improving freedom of movement or accessibility in the patient room (Autonomy scale). The Health Care Professionals working with their patients can often identify appropriate interventions once they know which of these domains a patient perceives as impaired.

On the other side, patients additionally could benefit from the regularly offered chance to take their mental health into account, feel seen and respected, and to express themselves.

### Strengths and limitations

A strength and limitation of this study is that it was conducted at a certified University Comprehensive Cancer Center. On the one hand, a University Comprehensive Cancer Center is supposed to provide care at the highest standards, provides treatment options that other institutions cannot offer, and treats both common and rare types of cancer. On the other hand, the high standards regarding the level of advanced training of health care professionals and equipment might not apply to smaller hospitals, especially in rural areas. This could lead to a bias in the study sample, their perceived HrQoL, and their response behavior. In summary, the results on the cut-offs for the HELP-6 could be different in a less specialized hospital or another health care system. Especially, since the design of the questionnaire and the intended use for the cut-offs is to improve clinical practice, the application of these cut-offs should be verified in other settings.

Moreover, the handling of missing data in the study sample could lead to a biased analysis as nine cases were excluded from analysis. Further bias could have been introduced to the study sample regarding the observation that 124 eligible patients declined participation because of emotional distress. In addition to the discussion of the single center study design, recruitment in the included five oncology departments offers a limited sample of the heterogeneous cancer population since not every cancer entity or treatment might be represented here. However, we were able to include a variety of cancer types, treatments, and stages.

Furthermore, the skewed distribution in this study is a limitation, and the results pertaining to it should be interpreted with caution. Specifically, two of the six scales are affected by this non-normal distribution. We have addressed these results throughout the manuscript. Furthermore, we decided to use the cut-off values of established and validated questionnaires as external criteria to discriminate the two groups of patients with more or less HrQoL. We did not include expert opinions from patients or HCPs generated for example by focus groups or interviews. Expert opinion on a definition of the more and less HrQoL impaired groups could have provided additional guidance. At the same time, both methodological approaches of group discrimination are widely used in literature.

### Implications for psychosocial providers

The calculated cut-offs provide guidance for interpreting HELP-6 results. It is intended to help health care professionals decide whether to address test results with patients and whether to request consultation with psychologists, social workers, or other professionals. The now accessible cut-offs for the HELP-6 make this short instrument for routine monitoring even more convenient to use in clinical routine.

## Supplementary Information

Below is the link to the electronic supplementary material.

Supplementary Material 1

## Data Availability

The dataset and code supporting the conclusions of this article are available on the platform of the Open Science Framework (https://osf.io/kdj57/?view_only=85c8db4dc2934b17a7b62ac829ab5391).

## Abbreviations

HELP-6: Hamburg Inventory for Measuring Quality of Life in Oncological Patients
PROM: Patient-reported outcomes measurement
HrQoL: Health-related quality of life
HCP: Healthcare professionals
PHQ-4: Patient Health Questionnaire 4
PDI-G: Assessment of patients’ dignity in cancer care
FACT-G: Functional Assessment of Cancer Therapy – General
DT: Distress Thermometer
SF-8: Short Form-8 Health Survey
ROC: Receiver Operating Characteristics
AUC: Area Under the Curve
SD: Standard Deviation
