# Supplementary material for: When do we need to take action? Cut-off values for the hamburg inventory for measuring quality of life in oncological patients (HELP-6)
Source: J Patient Rep Outcomes. 2026 Sep 19;10:168. doi: 10.1186/s41687-026-01198-8 (PMC13598032; doi:10.1186/s41687-026-01198-8)
Supplement: Supplementary file 1 [file 41687_2026_1198_MOESM1_ESM.docx]

Table S1: frequency and percentages of missing values by variable (*N*=630 patients)

| **scale** | **missing data** | |
| --- | --- | --- |
|  | *n* | % |
| Emotional Health,  HELP-6 | 6 | 0.7 |
| Physical Ailment,  HELP-6 | 5 | 0.7 |
| Social Functionality,  HELP-6 | 3 | 0.5 |
| Autonomy,  HELP-6 | 7 | 1.1 |
| Dignity,  HELP-6 | 9 | 1.4 |
| Resources,  HELP-6 | 6 | 1.0 |
| Distress-Thermometer | 9 | 1.4 |
| Physical Well-Being,  Fact-G | 51 | 8.1 |
| Social Well-Being,  Fact-G | 74 | 11.7 |
| Autonomy,  PDI-G | 11 | 1.7 |
| Loss of Sense of Worth and Meaning,  PDI-G | 41 | 6.5 |
| Functional Well-Being,  Fact-G | 52 | 8.3 |

Table S2: sensitivity analysis of ROC analyses using expected-maximization algorithm to impute all missing data (*N*=630 patients)

| **HELP-6 scale** | **Comparative scale** | **Area under the Curve (95% CI)** | **Youden-Index** | **Optimal cutoff** | **Sensitivity (%)** | **Specificity (%)** | **Positive likely-hood ratio** | **Negative likely-hood ratio** | **Positive predictive value (%)** | **Negative predictive value (%)** |
| --- | --- | --- | --- | --- | --- | --- | --- | --- | --- | --- |
| **Emotional Health** | Distress scale,  *DT* | 0.81  (0.78-0.85) | 0.52 | ≥ 5 | 80% | 72% | 2.88 | 0.27 | 76 | 77 |
| **Physical Ailment** | Physical well-being,  *Fact-G* | 0.86  (0.82-0.89) | 0.56 | ≥ 6 | 80% | 76% | 3.39 | 0.27 | 94 | 47 |
| **Social Functionality** | Social well-being,  *Fact-G* | 0.81  (0.74-0.89) | 0.56 | ≥ 3 | 89% | 67% | 2.73 | 0.16 | 97 | 31 |
| **Autonomy** | Loss of autonomy,  *PDI-G* | 0.72  (0.68-0.76) | 0.33 | ≥ 5 | 55% | 77% | 2.43 | 0.58 | 53 | 79 |
| **Dignity** | Loss of sense  of worth and meaning,  *PDI-G* | 0.64  (0.54-0.75) | 0.27 | ≥ 3 | 38% | 88% | 3.34 | 0.70 | 18 | 96 |
| **Resources** | Functional well-being,  *Fact-G* | 0.75  (0.70-0.80) | 0.42 | ≥3 | 67% | 75% | 2.68 | 0.44 | 91 | 38 |
